# Optimizing Community-Based Hepatitis B and C Care for Engaging Housing-Insecure Individuals

**DOI:** 10.1177/10732748251374419

**Published:** 2025-09-03

**Authors:** Vanessa Schick, Abigail Grace, F. Tiffany Quan, Cathy Troisi, Jack Tsai

**Affiliations:** 1 School of Public Health, University of Texas Health Science Center at Houston, Houston, TX, USA

**Keywords:** liver diseases, housing, health services, homelessness, patient care, poverty, preventative medicine, primary prevention, public housing, community health workers

## Abstract

**Introduction:**

Hepatitis B and C (HBV/HCV) are bloodborne infections, with individuals who have histories of substance use and homelessness bearing a disproportionate risk. Long-standing difficulties in engaging these populations have made testing and treatment challenging. This retrospective observational study describes a community-based approach to HBV/HCV prevention and treatment, comparing the effectiveness of different engagement site types in reaching and engaging this high-need population.

**Methods:**

GRASSROOTS HEALTH was launched in 2018 to improve HBV/HCV care by providing on-site testing, HBV vaccination, treatment navigation, and adherence support across various housing and drop-in centers. Outcomes were tracked through REDCap and analyzed by engagement site.

**Results:**

GRASSROOTS HEALTH reached nearly 2000 clients, with the greatest needs for HCV treatment in drop-in centers and HBV vaccination in low-income/permanent supportive housing. All sites demonstrated a relatively high return on effort, as evidenced by the percentage of participants needing HBV vaccination or HCV/HBV treatment.

**Conclusion:**

Engaging individuals through housing and service centers effectively reached a high-need community, with findings suggesting that different engagement points may enhance outreach based on the primary focus (HCV treatment vs HBV vaccination).

## Plain Language Summary

What is already known on this topic? Individuals experiencing homelessness face a higher risk of hepatitis B and C (HBV/HCV). Community-engaged outreach may better reach this at-risk population. What is added by this report? This brief research report highlights a model that connected nearly 2000 Texans facing housing insecurity to HBV/HCV care, with two-thirds needing HBV/HCV services. Findings suggest a better return on effort (ROE) when HBV prevention is delivered in low-income and permanent supportive housing than in other settings, while HCV treatment is more needed in drop-in centers compared to other settings. What are the implications for cancer control? This community model of HBV/HCV care can be replicated in various settings. Targeting engagement sites based on specific goals (HBV vaccination or HCV treatment) can help practitioners maximize outreach to high-need populations aligned with those goals.

## Introduction

Hepatitis B and C (HBV/HCV) are leading causes of hepatocellular carcinoma (HCC), a primary form of liver cancer.^1^ Primary prevention of HCC is possible through behavior modification and vaccines for HBV, while secondary prevention is available through antiviral treatment intended to treat HBV and cure HCV.^2,3^ HBV/HCV are blood-borne infections with risk factors including intravenous drug use and nonprofessional tattoos; factors which may occur in conjunction with homelessness/ housing instability.^4^ As such, people experiencing homelessness are estimated to be disproportionately impacted by HBV/HCV,^5^ making them a priority population in national strategic planning of the U.S. Department of Health and Human Services with a goal to enhance cross-sector partnerships.^3^

This retrospective observational study utilizes program data to describe an interdisciplinary model of HBV/HCV prevention and treatment designed to meet housing-insecure populations ‘where they are’ through the delivery of services across several engagement/retention sites (low-income, permanent supportive, transitional housing, and drop-in centers). The model was implemented in Texas, which has the highest HCC incidence rate in the continental United States.^6,7^ This study describes the model, client characteristics, and compares engagement/retention sites to identify the best strategies to maximize reach to people with experiences of housing insecurity and homelessness (PEH).

## Methods

**Figure 1.**
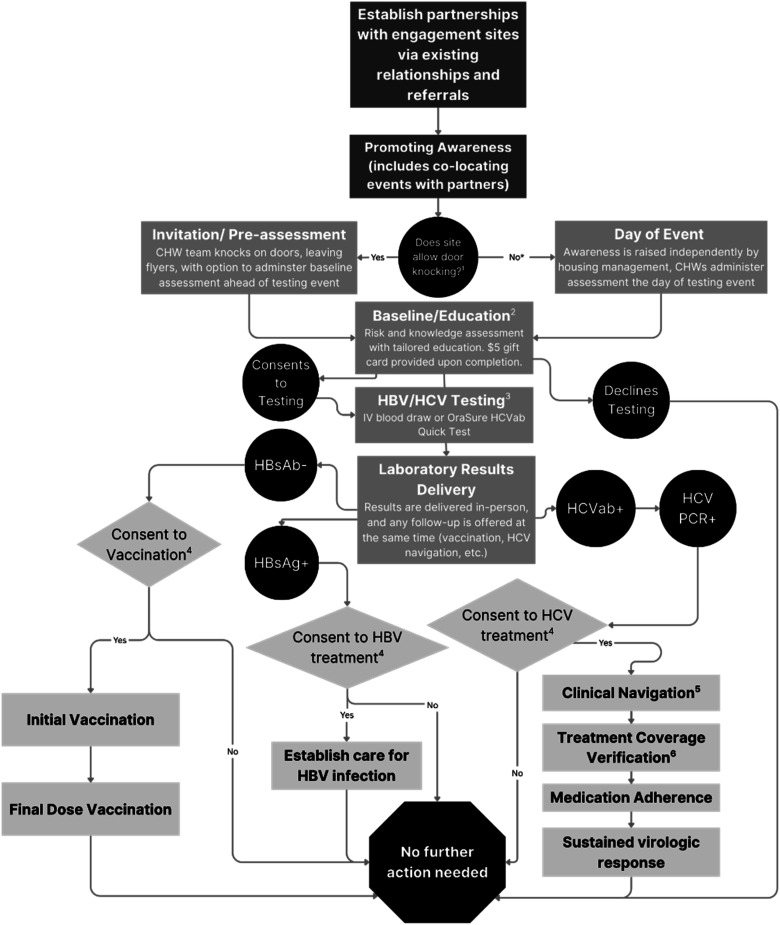
Workflow Diagram of GRASSROOTS HEALTH Service Model. (1) Some Housing Sites did not Permit Door Knocking or Preferred to Promote Testing Events Internally, Such as Through Mass Emails/Texts, Communication Through Case Managers, etc. (2) All Residents and Site Regulars (e.g., Staff or Guests With a Shared History) Aged 18 or Over Were Invited to Participate in the Baseline and Education Assessment. They Received the Gift Card Upon Completion of the Baseline Regardless of Their Testing Decision. (3) Everyone was Eligible for Testing unless They had Been Tested Within the Program Within the Past Year and Reported no new Risk Factors. All who Wanted to be Tested Were Offered the Opportunity. (4) All Eligible Clients Were Offered Vaccination and Treatment With Corresponding Education. Since the Goal is to Provide an Opportunity to Receive Services, all Client Decisions to Decline Services Were Respected. However, the Team never Considered a Decision ‘Final’ since Many Clients Modified Their Initial Decisions During Later Visits when Interacting With the GRASSROOTS HEALTH at Future Events. This may be due to Enhanced Self-efficacy, Reduced Barriers (e.g., Prison/Jail Sentences), or Enhanced Trust of the Team/Services Over Time. (5) Clients Were Given the Option to be navigated to Their Primary Care Provider (if One was Available) or a Clinical Partner for This Study (e.g., Healthcare for the Homeless Houston). The Clinical Partners Worked Closely With GRASSROOTS HEALTH and Service Costs Were Covered Through Clinical Programs (e.g., Funding to Serve Indigent Populations). (6) Uninsured Patients Were Treated Through Patient Assistance Programs while Insurance (e.g., Medicare, Medicaid) was Billed for Those With Proper Coverage.

Responding to a community need expressed through an existing community-academic partnership, GRASSROOTS HEALTH (03/2019-12/2024) was launched as a cross-sector collaboration to reduce barriers to the HBV/HCV continuum-of-care among PEH in Houston, Texas (4^th^ largest US. city) in 2019 with services extended to San Antonio (fourth and seventh largest U.S. cities, respectively) and surrounding counties in 2022. During visits to sites, all residents/attendees were invited to participate (convenience/consecutive sampling) by placing invitations on doors or engaging them in a shared space (see Figure 1). Community health workers (CHWs), trained/certified community liaisons with supplemental HBV/HCV training, administered a baseline HBV/HCV knowledge assessment and provided education. Participants received a $5 gift card upon completion, independent of testing decisions. Upon consent, 1-2 vacutainer tube(s) of venous blood were drawn and tested in a CLIA-certified laboratory for HBsAg for evidence of current infection and anti-HBs to determine immunity through infection/immunization. HCV reflex testing was used to determine HCV status. Clients unable to undergo venipuncture received a rapid HCV antibody test via fingerstick.

CHWs delivered HBV/HCV results in-person. Those who consented were vaccinated with Engerix-B® or HEPLISAV-B on the recommended schedule. Individuals who tested HBV/HCV positive were navigated to their primary care provider or a partnering clinic (e.g., federally qualified health clinic) for no/low-cost treatment. Partnering clinics were accessible via public transportation. CHWs scheduled appointments, accompanied clients to appointments, and coordinated/confirmed medications. Clients received three incentives ($50 total) for confirming treatment adherence.

### Measures

REDCap was used to record assessments and track client progress (e.g., testing, vaccination, treatment navigation). Navigation to healthcare was operationalized as 1+ clinical appointments. Engagement sites (N = 47) were categorized as low-income/affordable housing (U.S. Department of Housing and Urban Development [HUD] income requirements for Section 8 housing); permanent supported housing –PSH (HUD requirements for chronic homelessness vouchers); transitional housing (housing was time-limited); or drop-in center (housing variable including unsheltered populations). Return on effort (ROE) was operationalized as the percentage of clients with care needs, including active HBV/HCV or without HBV surface antibodies, relative to all clients tested. Chi-squared tests were used to assess initial significant associations between housing type and sociodemographic variables, and multiple logistic regressions were used to explore the association of housing type with various clinical outcomes, controlling for sociodemographic variables with significant bivariate associations. Pairwise deletion was used to handle missing data. Results were reported with chi^2^ test statistics, adjusted odds ratios, 95% confidence intervals (CI), and *P*-values. The UTHealth IRB determined data collection [HSC-SPH-18-0861-10/04/2018] and analysis were exempt [HSC-SPH-23-1058-11/29/2023] since data were not collected for research purposes and were deidentified before analysis. The reporting of this study conforms to STROBE guidelines.^8^

## Results

From 2018-2024, GRASSROOTS HEALTH screened 2073 clients, with site data for 1892 participants. Over half (54.23%, N = 1026) were aged 50-69, and the largest group identified as African American (46.41%, N = 878). Nearly one-third were Hispanic, and just over half were male. Engagement sites differed significantly by age, race, ethnicity, and gender (Table 1). PSH participants were older, mostly Black/African American and non-Hispanic, while low-income and drop-in center participants were predominantly White and Hispanic. The majority of participants in low-income housing were female, whereas other sites had a higher percentage of male participants.

**Table 1.** Characteristics of Clients Engaged in the Hepatitis B and C Continuum of Care Through the GRASSROOTS HEALTH Program by Community Engagement Sites (N = 1892)

	Total (N = 1892)		Low-income housing (N = 537)		Permanent supportive housing (N = 1112)		Transitional housing (N = 136)		Drop-in center (N = 107)		X2	*P*-value
	%	(N)	%	(N)	%	(N)	%	(N)	%	(N)		
Age											280.30	<0.001
18-29	5.7%	(107)	11.9%	(64)	2.3%	(26)	6.6%	(9)	7.5%	(8)		
30-39	13.8%	(261)	16.0%	(86)	9.6%	(107)	33.8%	(46)	20.6%	(22)		
40-49	17.4%	(330)	17.1%	(92)	14.2%	(158)	25.7%	(35)	42.1%	(45)		
50-59	29.1%	(551)	19.4%	(104)	36.0%	(400)	19.9%	(27)	18.7%	(20)		
60-69	25.1%	(475)	21.6%	(116)	30.1%	(335)	9.6%	(13)	10.3%	(11)		
70+	8.7%	(164)	14.0%	(75)	7.4%	(82)	4.4%	(6)	0.9%	(1)		
Unknown	0.2%	(4)	0.0%	(0)	0.4%	(4)	0.00%	(0)	0.00%	(0)		
Race											412.03	<0.001
American Indian/Alaska native	1.1%	(21)	0.7%	(4)	1.2%	(13)	1.5%	(2)	1.9%	(2)		
Asian	1.2%	(22)	2.6%	(14)	0.5%	(6)	0.00%	(0)	1.9%	(2)		
Native Hawaiian/Pacific Islander	0.1%	(1)	0.0%	(0)	0.10%	(1)	0.00%	(0)	0.00%	(0)		
Black or African American	46.4%	(878)	25.3%	(136)	63.9%	(711)	13.%	(18)	12.2%	(13)		
White	39.7%	(751)	56.8%	(305)	25.5%	(284)	68.4%	(93)	64.5%	(69)		
More than one race	6.3%	(119)	5.0%	(27)	4.8%	(53)	14.7%	(20)	17.8%	(19)		
Unknown	5.3%	(100)	9.5%	(51)	3.9%	(44)	2.2%	(3)	1.9%	(2)		
Ethnicity											508.33	<0.001
Hispanic	30.6%	(579)	61.8%	(332)	11.2%	(125)	41.9%	(57)	60.8%	(65)		
Not hispanic	67.8%	(1282)	35.8%	(192)	87.2%	(970)	58.1%	(79)	38.3%	(41)		
Unknown	1.6%	(31)	2.4%	(13)	1.3%	(17)	0.00%	(0)	0.9%	(1)		
Gender											265.24	<0.001
Female	42.3%	(800)	69.7%	(374)	28.4%	(316)	45.6%	(62)	44.9%	(48)		
Male	56.0%	(1059)	27.9%	(150)	69.8%	(776)	54.4%	(74)	55.1%	(59)		
Another response	0.5%	(10)	0.4%	(4)	0.5%	(6)	0.0%	(0)	0.0%	(0)		
Unknown	1.2%	(23)	1.7%	(9)	1.3%	(14)	0.0%	(0)	0.0%	(0)		

**Figure 2.**
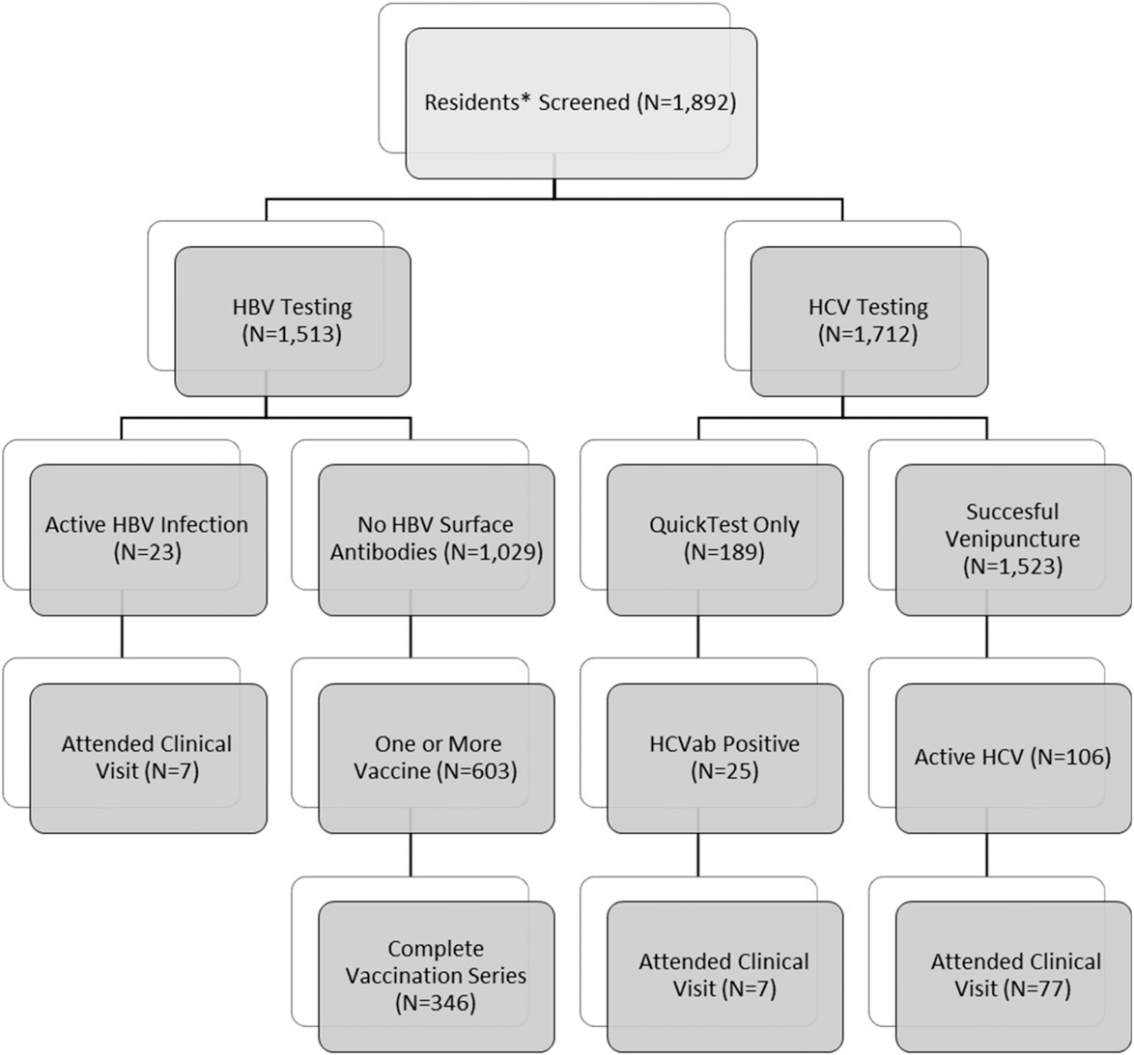
Flow Diagram of the Number of Residents Engaged in the HBV/HCV Continuum of Care Through the GRASSROOTS HEALTH Program. Note. Data on the Reasons for Drop-Out From Screening to Testing Were Only Collected in Phase 2 Clients (N = 1191). A Total of 2.35% Refused/were not Interested. Another 4.11% Were not Home or Unavailable (N = 49) With 8.75% of Low-Income Housing Sites Being Unavailable due to the Fact that the Screener was Conducted in Advance of the Blood Draw at Some Housing Sites

Over 1500 participants were tested for HBV (Figure 2), with 1.52% (N = 23) having an active infection. Most (68.01%, N = 1029) had no HBV surface antibodies, and of those, 58.60% (N = 603) received vaccination, with 346 completing the series. Nearly 1700 participants were tested for HCV, with 6.96% (N = 106) having an active infection. Among these, 72.65% (N = 77) attended an HCV appointment, compared to 30.43% (N = 7) of HBV-positive clients.

Accounting for significant demographic characteristics, participants at drop-in centers were twice as likely to report prior HBV testing relative to those in low-income housing (AOR = 2.03, *P* < .001), with those in low-income housing significantly less likely to report HCV testing relative to all other engagement sites (Table 2). Upon testing, those in transitional housing and drop-in centers were over 1.5x times more likely to have HBV surface antibodies relative to those in low-income housing, a difference not explained by active HBV infection. Similarly, the likelihood of engaging an HCV antibody-positive client was significantly higher at all sites compared to low-income housing, with odds ranging from 5.73 (AOR = 5.73, *P* < .001) to 12.6 (AOR = 12.6, *P* < .001). This pattern also extended to active HCV infection. Overall, ROE (those with a care need, including active HBV/HCV or without HBV surface antibodies) was high (>58%), peaking at 72.5% in permanent supportive housing.

**Table 2.** GRASSROOTS HEALTH Clients Hepatitis B and C Self-Reported Testing History and Infection Status by Community Engagement Site (N = 1892)

	Previously tested for HBV^a^				Previously tested for HCV^a^				Active HBV	HBV surface antibodies				HCVab positive				Active HCV	Return on effort			
	%^b^	AOR	*P*-value	95% CI	%^b^	AOR	*P*-value	95% CI	%^b^	%^b^	AOR	*P*-value	95% CI	%^b^	AOR	*P*-value	95% CI	%^b^	%^b^	AOR	*P*-value	95% CI
Low-income housing (N = 537)	35.9%	Ref	Ref	Ref	32.0%	Ref	Ref	Ref	0.7%	28.8%	Ref	Ref	Ref	6.1%	Ref	Ref	Ref	1.6%	71.9%	Ref	Ref	Ref
Permanent supportive housing (N = 1112)	48.3%	1.2	0.21	0.88-1.76	50.3%	1.6	0.02*	1.11-2.26	2.4%	30.3%	1.4	0.11	0.93-2.03	23.3%	5.7	0.00***	3.11-10.56	8.0%	72.5%	0.82	0.27	0.58-1.16
Transitional housing (N = 136)	46.7%	1.3	0.18	0.87-2.07	44.5%	1.8	0.03*	1.13-2.71	0.0%	47.3%	1.8	0.01*	1.13-2.90	24.0%	9.2	0.00***	4.61-18.24	10.1%	58.1%	1.08	0.72	0.70-1.68
Drop-in center (N = 107)	56.1%	2	0.00***	1.28-3.22	63.6%	3.5	0.00***	2.18-5.64	0.0%	41.2%	1.7	0.05*	1.01-2.81	33.0%	13	0.00***	6.29-25.23	19.6%	69.1%	1.22	0.42	0.76-1.96

All models included the following control variables significant at the bivariate level: Age Group, Race, Ethnicity, Gender, and Veteran status. Some of the models (Active HBV and Active HCV) had perfect prediction on some levels due to small cell sizes, and were unable to be run. Return on Effort is operationalized as the % of individuals screened who did NOT have HBV surface antibodies or had active HBV or HCV out of the total number screened.

*P < .05, **P,.01, ***P < .001.

Active HBV and HCV did not have a sufficient sample size to analyze differences between sites.

^a^Previous testing for HBV and HCV is based on client self-report.

^b^Percentages calculated based on the incidence of each outcome over total sample size of each housing type.

## Discussion

With nearly 2000 clients served, GRASSROOTS HEALTH potentially demonstrates an effective community-based model for engaging high-need individuals in the HBV/HCV continuum of care with HBV/HCV prevalence rates exceeding national (.4% HBV) and state-level estimates (1.79% HCV).^9,10^ Compared to a traditional outreach program in the same region, GRASSROOTS HEALTH’s targeted approach of meeting clients in high-priority living/gathering areas reached more high-need clients (1.3% vs 6-33%) and achieved markedly higher care navigation success.^11^ Nearly 60% of HBV-negative participants received at least one HBV vaccination, and 73% of HCV-positive participants were navigated into treatment. Fewer HBV-positive clients were navigated due to logistical factors (e.g., disproportionate HBV during COVID-19) and motivational factors since HBV is treated and not cured.

Client demographics differed across engagement sites, with higher proportions of men at sites targeting chronic homelessness and substance use. Drop-in center clients were the most likely to report prior HBV/HCV testing, possibly due to their higher risk, as some specifically served individuals engaged in injection drug use and may have prioritized HCV testing. Over 5 times fewer clients were HCV-positive in low-income housing relative to other engagement sites, possibly due to their homelessness and risk history. Nearly half of those testing positive had active infections, indicating gaps in HCV care. Prioritizing drop-in centers, transitional housing, and PSH could maximize the reach of those with HCV-related needs.

In the US, outside of HBV prenatal testing requirements, HBV/HCV testing is not required. Most states have required childhood vaccination for ∼25 years and recommend adult vaccination, yet adult coverage remains around 30%.^10,12^ This is consistent with the client-detected rate with lower HBV surface antibodies in permanent supportive and low-income housing relative to transitional housing and drop-in centers. Without anti-HBc antibodies, it is unclear if this is due to differences in risk exposure or vaccination rates. These findings suggest reduced HBV immunity among low-income and PSH residents, highlighting an opportunity to expand prevention efforts, including vaccination within these communities.

Return on effort (ROE) illustrates how many people screened are actually in need of HBV/HCV service(s). The relatively high ROE (58-72%) detected suggests the model targeted the high-need individuals. Maximizing the impact of each dollar spent may be increasingly critical in tightening funding environments. Engagement sites showed similar returns, with sites varying in HBV prevention and HCV treatment opportunities. PSH maximized both while providing the extra benefit of a stable place for long-term engagement.

This study had several limitations, including convenience sampling and self-reported testing history. Results may reflect differences in individuals willing to be tested rather than the broader population at each site. Additionally, data were collected across multiple years and cities, introducing potential historical and regional variations in testing and outcomes. Future research should examine differences in vaccination rates and treatment retention across diverse persons (e.g., country of birth) and site types, as housing stability may influence long-term care engagement.

### Conclusions

This study describes a successful interdisciplinary community model for engaging and treating an underserved, high-need population at risk of HBV/HCV. This model targeted areas with concentrated populations of high-risk individuals, delivering care directly in nonclinical settings (e.g., housing). This strategy yielded a high return-on-effort by engaging many individuals in need at each site, outperforming conventional outreach methods. Data on differences in HBV/HCV outcomes between sites provides insight on where to target efforts to differentially reach the communities in greatest need of HBV/HCV prevention and treatment.
